# Novel bioinformatic developments for exome sequencing

**DOI:** 10.1007/s00439-016-1658-6

**Published:** 2016-04-13

**Authors:** Stefan H. Lelieveld, Joris A. Veltman, Christian Gilissen

**Affiliations:** Department of Human Genetics, Radboud Institute for Molecular Life Sciences, Radboud University Medical Center, Geert Grooteplein 10, 6525 GA Nijmegen, The Netherlands; Department of Human Genetics, Donders Centre for Neuroscience, Radboudumc, Geert Grooteplein 10, 6525 GA Nijmegen, The Netherlands; Department of Clinical Genetics, GROW-School for Oncology and Developmental Biology, Maastricht University Medical Centre, Universiteitssingel 50, 6229 ER Maastricht, The Netherlands

## Abstract

With the widespread adoption of next generation sequencing technologies by the genetics community and the rapid decrease in costs per base, exome sequencing has become a standard within the repertoire of genetic experiments for both research and diagnostics. Although bioinformatics now offers standard solutions for the analysis of exome sequencing data, many challenges still remain; especially the increasing scale at which exome data are now being generated has given rise to novel challenges in how to efficiently store, analyze and interpret exome data of this magnitude. In this review we discuss some of the recent developments in bioinformatics for exome sequencing and the directions that this is taking us to. With these developments, exome sequencing is paving the way for the next big challenge, the application of whole genome sequencing.

## Introduction

Bioinformatics has been central to the analysis and interpretation of exome sequencing data. Initial bioinformatics challenges concerned quality control; short read-mapping (Langmead et al. 2009; Li and Durbin 2009), variant calling (Albers et al. 2011; Li et al. 2009; McKenna et al. 2010), and variant annotation (Jager et al. 2014; Liu et al. 2013; Ng et al. 2009; Yang and Wang 2015). Most of these challenges have now been tackled to a degree that bioinformatic workflows are available to analyze and interpret exomes in a standard fashion and provide workable results (DePristo et al. 2011; Pabinger et al. 2014). Some of the original hurdles have simply become less relevant with the progression of technology giving rise to more and higher quality sequence data and longer sequence reads (e.g., the ambiguous alignment of very short sequencing reads). Nevertheless, quality control of exome sequencing data still remains a necessity to guarantee reliable downstream results. This task has now become fairly routine through the development of several software packages that facilitate the assessment of standard quality control measures for exome sequencing (Li et al. 2009; McKenna et al. 2010; Okonechnikov et al. 2016; Quinlan and Hall 2010).

With the widespread adoption of next generation sequencing (NGS) technologies by the genetics community and the rapid decrease in costs per base, exome sequencing has become a standard within the repertoire of genetic experiments for both research and diagnostics (Neveling et al. 2013; Yang et al. 2013). Although whole genome sequencing represents the ultimate genetic experiment, exomes still offer advantages in terms of costs, speed and ease of data storage and analysis. The steady increase of sequencing capacity and the widespread application of exome sequencing has allowed the sequencing of thousands of individuals and studies with hundred thousands of exomes are already in progress (Fu et al. 2013; Lohmueller et al. 2013; The Deciphering Developmental Disorders Study 2015; Walter et al. 2015). As an example, the Exome Aggregation Consortium (ExAC) collected a dataset of over 60,000 individuals and will grow even larger in the nearby future (Lek et al. 2015). This scale at which exome data are now being generated has given rise to novel challenges in bioinformatics to store, analyze and interpret exome data of this magnitude (Stephens et al. 2015). In this review we will discuss some of the recent developments in bioinformatics for exome sequencing. We have summarized some of the tools that we believe may be of interest to the reader in Table 1.

**Table 1:** Overview of some of the novel bioinformatics tools related to the storage, analysis or interpretation of exome sequencing data

Name	Description	Website
Data-compression		
CRAMtools	Framework to compress BAM files into CRAM format	https://github.com/enasequence/cramtools
Scramble	C implementation of CRAM to compress BAM into CRAM format for faster encoding	http://sourceforge.net/projects/staden/files/io_lib/
TABIX	Tool to index and query bgzip-compressed VCF formatted files, available via SAMtools	http://sourceforge.net/projects/samtools/files/tabix/
Genotype query tools	Toolset to compress and query VCF files. Designed to compress large-scale cohorts	https://github.com/ryanlayer/gqt
Cloud tools		
CloudBurst	Cloud-based parallel read-mapping algorithm to map sequence reads to a reference	http://sourceforge.net/projects/cloudburst-bio/
Cloud aligner	Cloud-based Hadoop MapReduce-based approach mapping of sequence reads	http://cloudaligner.sourceforge.net/
Crossbow	Cloud-computing software tool that combines read-mapping and the SNP genotyping	http://bowtie-bio.sourceforge.net/crossbow/index.shtml
VAT	Variant Annotation Tool (VAT) is a Cloud-based platform to functionally annotate variants	http://vat.gersteinlab.org/
Mercury	A whole exome sequencing analysis workflow deployed In the Amazon Web Services (AWS) cloud	https://www.hgsc.bcm.edu/software/mercury
Variant prioritization tools		
CADD	Combined 63 annotations into one meta-score (C score) for the entire genome based on a SVM	http://cadd.gs.washington.edu/
Eigen	Spectral approach to the functional annotation of genetic variants in coding and non-coding regions.	http://www.columbia.edu/~ii2135/eigen.html
DANN	DANN used the same feature set and training data as CADD to train a deep neural network (DNN).	https://cbcl.ics.uci.edu/public_data/DANN/
FitCons	Predictions of pathogenicity for the entire genome based on evolutionary conservation and functional data	http://compgen.cshl.edu/fitCons/
SPANR/SPIDEX	Trained a model optimized for the prioritization of splice site variants with a deep learning approach	http://www.deepgenomics.com/spidex
HAL	Prioritization of splice site variants based on their effect of (alternative) RNA splicing	http://splicing.cs.washington.edu
PHIVE	Analysis of exome variants by computing phenotype similarity between human disease phenotypes and phenotype information from knockout experiments in model organisms	http://www.sanger.ac.uk/resources/databases/exomiser
RVIS	The Residual Variation Intolerance Score or RVIS is a gene based score to prioritize disease genes based on intolerant to genetic variation	http://genic-intolerance.org/
CNV detection		
CoNIFER	Detects rare CNVs in exome data based on sequence read-depth	http://conifer.sourceforge.net/
XHMM	Uses principal-component analysis (PCA) to normalize exome read-depth and a hidden Markov model (HMM) to detect CNVs	https://atgu.mgh.harvard.edu/xhmm/
Codex	Normalization and CNV calling procedure for whole exome sequencing data	http://www.bioconductor.org/packages/devel/bioc/html/CODEX.html
Data sharing		
ExAC	60,706 unrelated individuals sequenced as part of various disease-specific and population genetic studies	http://exac.broadinstitute.org/
DECIPHER	Database containing data from 18,533 patients who have given consent for broad data-sharing	https://decipher.sanger.ac.uk/
Café variome	Platform to share genetic variant and phenotype data on a global scale	http://www.cafevariome.org/
GeneMatcher	Online platform designed to connect clinicians and researchers from around the world who share an interest in the same gene or genes	https://genematcher.org/
RD-connect	Platform that links up data used in rare disease research into a central resource for researchers worldwide	http://rd-connect.eu/
PhenomeCentral	Repository for secure data-sharing targeted to clinicians and scientists working in the rare disorder community	https://www.phenomecentral.org/
MatchMaker Exchange	Platform enabling matching of cases with similar phenotypic and genotypic profiles though a number of databases	http://www.matchmakerexchange.org/
Phenotypes		
Phenotips	A software tool for collecting and analyzing phenotypic information for patients with genetic disorders	https://phenotips.org/
PhenoDB	A software tool to store and analyze standardized phenotypic information	http://phenodb.net
Phenominer	A tool to extract structured phenotypes from text	http://phenominer.mml.cam.ac.uk/

## More data, more storage

With growing datasets, simply storing data becomes a challenge that all laboratories will at some point need to face. Sequencing instruments typically generate FASTQ files containing all individual sequencing reads. After alignment the resulting reads are stored in the Sequence Alignment/Map (SAM) format that describes where sequence reads are mapped onto the reference genome. SAM files are usually compressed into the binary SAM (BAM) format that reduces the file size 3–4 times (Li et al. 2009). The BAM format is currently the de facto standard format for aligned reads and can be used by a large variety of downstream analysis and visualization tools (Li et al. 2009; Quinlan and Hall 2010; Thorvaldsdottir et al. 2013). Genomic variants that are subsequently identified based on the BAM file are then stored in a variants call format (VCF) (Danecek et al. 2011). The typical size of a single exome BAM file is within the range of Gigabytes whereas the VCF file is usually no more than 100 MB.

### Storing less

The most straightforward method for reducing data storage needs is by simply storing less data, or by removing data as soon as possible. As an example, sequencing instruments currently only store raw images of the sequencing process for a limited time for trouble-shooting after which they are discarded. Similarly, many labs no longer keep the original raw sequencing reads (FASTQ file) after alignment since modern sequence aligners also include reads in the BAM file that are not aligned to the genome. This adds a little bit to the size of the BAM files but there is no longer any need for storing FASTQ files, since raw reads can now be regenerated from the alignment files by tools like Picard (http://picard.sourceforge.net.) and SAMtools (Li et al. 2009). This potentially reduces storage requirements by half. In addition to this, several clinical guidelines have been proposed that allow diagnostic laboratories to remove the alignment files after 1 or 2 years (Rehm et al. 2013; Weiss et al. 2013). However, although VCF files contain the primary result of the experiment it is worthwhile to keep BAM files for future analysis since they contain much more information than VCF files, for example the identification of CNVs (Krumm et al. 2012), somatic mutations (Lindhurst et al. 2011), and mitochondrial DNA variation (Samuels et al. 2013). It is not uncommon that reanalysis of FASTQ or BAM files can identify additional variants that were initially missed (Zighelboim et al. 2014).

### Compression

An alternative to the straightforward removal of large files to save space is data-compression. This has already been introduced for raw sequence files that are now by default compressed with gzip.

Although the SAM/BAM format is convenient in the sense that it contains almost all information of the original reads and all details about the alignment in an intuitive fashion, it was not designed for efficient storage (Li et al. 2009). Since BAM files are already in binary format, ordinary compression algorithms cannot significantly reduce their size. However, specialized compression tools use various techniques to further reduce the size of BAM files. First of all non-essential information, e.g. read identifiers, can be removed. Secondly, the majority of the exome will be the same as the reference genome and can be stored more efficiently: Reference-based compression encodes reads based on a reference sequence and stores only positions that differ from the reference sequencing (Hsi-Yang Fritz et al. 2011; Kingsford and Patro 2015). For regions where there are no differences to the reference genome, only coordinates and depth information are retained. Lastly, individual base quality scores (or Q scores) are typically encoded as PHRED-like scores within a range of 0–40 (Ewing and Green 1998). These quality scores are used to optimize read-mapping and variant calling. However, the scale of quality scores is very fine-grained and encoding Q scores into bins reduces storage space (Hach et al. 2012; Ochoa et al. 2013). Binning quality scores often results in compression with some loss of information (lossy compression), where the original quality scores lose precision during compression. The lost precision does, however, not necessarily result in significant loss of accuracy for variant calling (Yu et al. 2015).

Based on these approaches, alternative formats such as Goby (Campagne et al. 2013), SlimGene (Kozanitis et al. 2011), CRAM (Hsi-Yang Fritz et al. 2011) and DEEZ (Hach et al. 2014), have been introduced that attempt to keep as much of the original information yet at a lower cost of disk space than BAM. In particular, the CRAM format has gained a lot of traction. Compression of a BAM file to CRAM format with the Scramble tool resulted in file reductions of 38–55 % with a compression time of a few minutes (Bonfield 2014). CRAM compression has already been applied to tackle storage-capacity problems in large databases such as Sequence Read Archive (SRA) (Cochrane et al. 2011) and the 1000 Genomes project (http://www.1000genomes.org). The CRAM format is now supported by some widely used genomic analysis tools such as SAMtools, Picard and GATK (Li et al. 2009; McKenna et al. 2010). With the increasing support for the CRAM format, it may well replace the use of BAM files in the near future.

With ever growing datasets containing variants of thousands of individuals, it becomes worthwhile to compress the relatively small VCF files as well. The Tabix format offers a convenient compression format for large VCF files. This reduces file sizes roughly 3–5 times, and also supports indexing to perform efficient querying of genome positions (Li 2011). Some common resources are already available in Tabix format such as dbSNP (NCBI Resource Coordinators 2015) and Combined Annotation-Depended Depletion (CADD) scores (Kircher et al. 2014). Another option is to use the recently published Genotype Query Tools (GQT) to index and compress large number of VCF files. This tool also facilitates fast querying. GQT was used to compress the Exome Aggregation Consortium (ExAC) VCF file, consisting of 9.36 million exonic variants for 60,706 individuals, from 14.1 TB to 28 GB (Layer et al. 2016).

All in all, the growing need to reduced storage space is leading to new data formats for alignment and variant files and smarter algorithms to query these efficiently.

### Cloud-based solutions

Compression of data is an easy and efficient way to reduce storage needs, but in the end the reduction in data sizes is limited. An alternative is to store large amounts of genomics data in the cloud. Cloud storage offers several out-of-the-box advantages to local storage: it is scalable, has default access control policies, protects against data loss, allows for auditing, data encryption, easy sharing, and automation by programmable interfaces (Fusaro et al. 2011; Stein 2010). Currently, there are multiple commercial providers of cloud services of which Amazon Web Services (AWS; https://aws.amazon.com/), Microsoft Azure (https://azure.microsoft.com) and Google cloud platform (https://cloud.google.com) are the largest. In addition there are non-profit organizations offering cloud-computing solutions such as Open Cloud Consortium (http://occ-data.org/).

Cloud storage is based on a “pay as you go” monetary model whereby one only pays for used storage that can be expanded ad hoc. Although cloud storage itself is relatively inexpensive with less than $100 for storing 1 TB of data per month, there are some additional costs to consider (Shanahan et al. 2014). While transferring data into the cloud storage is usually free of costs, analyzing and downloading data from the cloud can be relatively expensive. For example, downloading 1 TB of data from the cloud costs approximately $120 per TB (Shanahan et al. 2014). This makes it worthwhile not only to keep data in the cloud but also to perform the analysis there and only download smaller result files. Special software is, however, needed to make efficient use of the scalability of the cloud-computing platform. Currently there are already a variety of tools for cloud-based mapping of sequence reads, (Nguyen et al. 2011; Schatz 2009), genotyping (Gurtowski et al. 2012), variant annotation (Habegger et al. 2012) as well as complete cloud-based exome sequencing pipelines (Liu et al. 2014; Reid et al. 2014). Fusaro et al. showed that the alignment of the entire genome (4 billion paired reads, 35 pb long) of a person in 48 h costing approximately $48 of cloud resources (Fusaro et al. 2011). According to Stein et al. the International Cancer Genome Consortium (ICGC) analyzed 500 genomes in the cloud for a price of $18 per sample whereas the authors estimate this would require $200 on standard computer systems (Stein et al. 2015).

Data stored in the cloud can also provide a solution for effective public and private data-sharing. For example, the Amazon Web Services (AWS) contains 1000 Genomes Project data (Clarke et al. 2012) and accommodates 1200 whole genome sequences of the ICGC. In addition, data from Ensembl and GenBank are being hosted in AWS and data transfer between AWS instances is free of charge (Fusaro et al. 2011). Furthermore, the US National Cancer Institute is exploring how the cloud could facilitate a cost-effective platform to store and share large amounts of tumor data (https://cbiit.nci.nih.gov/ncip/nci-cancer-genomics-cloud-pilots).

The uptake of cloud-based solutions by academic and non-academic hospital laboratories has been slow, likely because of practical concerns, unfamiliarity, as well as ethical and legal concerns of storing patient DNA data in the cloud (Dove et al. 2015). Although data storage in the cloud is relatively inexpensive, transferring vast quantities of sequencing data via the Internet from and into the cloud may be a considerable cost and a time-consuming process due to low network bandwidth (Schatz et al. 2010; Stein 2010). In addition, moving genetic data of patients to a third-party server introduces issues concerning security and privacy (Greenbaum et al. 2011). This has limited the use of cloud-based storage solutions for most clinical NGS applications so far. However, given the advantages and a future of routine genome sequencing, it may well be unavoidable that all genomics data end up in the cloud for analysis and for patients and their physicians to access.

## Variant identification

To some extent, challenges for calling variants have become less urgent with improved exome enrichment assays, increasing sequence quality and read length and reduced sequencing prices, allowing for higher coverage sequencing of the exome in individual patients. Whereas early comparisons of whole exome capture kits showed that around 80 % of the human protein coding sequence regions were captured at a minimal coverage of 20× (Parla et al. 2011), current exome capture kits and sequencing at minimal 100× median average coverage capture more than 95 % of the coding regions with a minimal coverage of 20× (Lelieveld et al. 2015). Due to the increased coverage and improved sequencing quality for modern exomes, variant calling has become more reliable. Several studies have even demonstrated the identification of somatic mutations for rare syndromes (Lindhurst et al. 2011; Poduri et al. 2013; Sato et al. 2014), which is only possible with high coverage. These improvements in exome quality have led some laboratories to reconsider the validation of sequencing variants by the gold standard Sanger sequencing. A recent validation study found that all single nucleotide variants with a Genome Analysis Toolkit (GATK) (McKenna et al. 2010) quality score above 500 were confirmed by Sanger sequencing and estimated that only validating variants with a quality score below this threshold would reduce the Sanger confirmation workload by 70–80 % (Strom et al. 2014). Overall the significant improvements in exome sequencing quality may indeed eliminate the need for validation of high quality variants. However, the detection of SNVs in NGS data has not been fully resolved and results from different variant callers remain inconsistent (O’Rawe et al. 2013; Pabinger et al. 2014; Zook et al. 2014). In addition, small insertions/deletion (indels) are still particularly problematic to identify accurately (Jiang et al. 2015b). Highly accurate genotypes across the genome of a single individual as for example provided by the “Genome in a Bottle Consortium” may help resolve these issues in the future (Zook et al. 2014).

### Detection of copy number variants

From SNVs attention has moved towards the identification of other types of variants in exome sequencing data. In particular, the identification of copy number variation (CNV) from exome data poses an attractive possibility as CNVs are an important cause of disease (Zhang et al. 2009). Genomic microarray platforms such as the SNP-array and Array CGH are the de facto standard to detect CNVs (Miller et al. 2010), whereas whole genome sequencing will likely be the preferred platform for the detection and characterization of CNVs as well as other structural variants (Gilissen et al. 2014). In contrast to microarrays and whole genome sequencing, exome sequencing targets only 1–2 % of the protein coding regions of the genome. The sparse and fragmented nature of exome data makes it more difficult to identify CNVs and methods rely mostly on depth-of-coverage approaches. For these approaches a normalized read count in a genomic window of a single individual is compared to that of other exomes. Normalization of read counts is required to counteract technical issues such as poor read mappability, GC bias, and batch effects between sequencing experiments (Teo et al. 2012). Many different algorithms have been devised based on read-depth methods, such as CODEX, Convex, Conifer, and XHMM (Amarasinghe et al. 2013; Fromer et al. 2012; Jiang et al. 2015a; Krumm et al. 2012). Comparisons of CNV algorithms for exome data have shown that none of the algorithms performed well in all situations and that the resolution is limited to at least three exome targets (de Ligt et al. 2013; Fromer et al. 2012; Krumm et al. 2012). Although this does not equal the sensitivity of high-resolution microarrays, it is comparable to that of medium resolution microarrays that are still commonly used. Studies describing the large-scale application of CNV detection only based from exome data are, however, still limited (Poultney et al. 2013), which may perhaps hint at some of the underlying difficulties to obtain robust CNV calls from exome data. The possibility to detect copy number variants in exome data is, however, a great benefit of exome sequencing that should not be ignored as CNVs contribute significantly to disease. The identification of other types of structural variants such as inversions, and the accurate prediction of CNV breakpoint remains challenging and whole genome sequencing is likely needed for this (Meienberg et al. 2015).

## Variant interpretation

Sequencing the protein coding regions of a patient typically yields tens of thousands of variants of which the majority is likely to be benign and only one or perhaps two variants contribute to the disease phenotype (Bamshad et al. 2011; Gilissen et al. 2012). The most effective way of distinguishing benign from pathogenic variants is based on using population frequencies of variants. For this approach all variants occurring in the population at higher frequencies than the disease prevalence are considered as benign. Databases with collections of exome variants of individuals without clear disease phenotypes have therefore been tremendously helpful to prioritize variants in Mendelian disease. This has given rise to several initiatives for large-scale variant databases with exome data (Fu et al. 2013; Tryka et al. 2014). The largest of these, thus far, is the Exome Aggregation Consortium (ExAC) database, containing variants of more than 60,000 exomes (Lek et al. 2015). These large databases are instrumental to the interpretation of future exomes for Mendelian disease gene identification. In addition, a need for population-specific databases of variation will remain, especially for those populations that are poorly represented in the large public databases (Tennessen et al. 2012). Interpretation based on population frequency information from databases should be done with care because of the possibility of false positives (MacArthur and Tyler-Smith 2010), founder mutations (Gunel et al. 1996), somatic or tissue-specific variants (Acuna-Hidalgo et al. 2015).

### Coding mutations

Although accurate population frequencies are a necessity for the interpretation of exomes, this will only reduce the number of possible candidate mutations to a couple of hundred or so (Gilissen et al. 2012). Further prioritization of pathogenic variants remains a challenging task, in particular for missense variants. Various tools such as Polyphen2 (Adzhubei et al. 2013), SIFT (Kumar et al. 2009) and PhyloP (Pollard et al. 2010), have long been used in the pathogenicity assessment of these protein coding variants based on evolutionary conservation. Unfortunately, these prioritization methods lack specificity and sensitivity to sufficiently reduce the large number of candidate mutations from exome sequencing on their own (Gilissen et al. 2012). This becomes even more apparent when considering the prioritization of non-coding variation from whole genome sequencing experiments.

However, in the last few years, novel tools have been published that are expected to offer better sensitivity and specificity compared to the traditional prioritization tools. The availability of genome-wide functional annotations of coding and non-coding variants in combination with algorithmic improvements resulted in novel tools adapted to prioritize both coding and non-coding variants. These novel tools can broadly be divided into two groups. The first group focuses on the prediction of deleterious variation by computing a functional meta-score based on integrating a variety of genome-wide annotations. Combined Annotation-Depended Depletion (CADD) is the most well-known example of such a framework that applies a support vector machine (SVM) to integrate 63 sources of functional and evolutionary data into a relative pathogenicity score (Kircher et al. 2014). Eigen (Ionita-Laza et al. 2016) and DANN (Quang et al. 2015) are other examples using different algorithmic approaches to combine large varieties of annotations into one pre-computed meta-score trained to distinguish between benign and deleterious variants. Fitness consequence (FitCons) (Gulko et al. 2015) is different in the sense that it compares patterns of divergence between the human population and primates to assess functional sites that emerged quite recently. The second group of tools attempts to specifically predict non-coding regulatory variants. DeepSEA (Zhou and Troyanskaya 2015) and DeltaSVM (Lee et al. 2015) are examples of such tools and were trained on a variety of annotations of non-coding annotations mainly derived from the ENCODE project (The ENCODE Project Consortium 2012). Notably, the DeepSEA method was based on a Deep learning algorithm, which is a form of machine learning that is increasingly being applied to biological problems (Alipanahi et al. 2015; Rusk 2016).

In spite of the potential of these tools, it remains unclear how well they perform in clinical practice because independent validation studies for these novel tools are still lacking. Moreover, such studies are hampered by a lack of sufficient validation data that have not already been used in the development of the prediction software or original benchmark (Grimm et al. 2015). Van der Velde et al. demonstrated the practical utility of CADD for the interpretation of variants. The authors applied CADD to a set of 2210 variants that were manually assessed by an expert panel and found that, beside a relatively small number of discrepancies in favor of the expert, CADD scores proved valuable for the prioritization of pathogenic variants. (van der Velde et al. 2015). However, a recent validation of CADD and other prediction tools using in vivo mouse models, found that about half of the assessed mutations that were predicted to be deleterious had little impact on the clinical phenotype (Miosge et al. 2015). This once again highlights the importance of functional validation of potential pathogenic variants.

### Splice site mutations

Due to the increased read lengths, exome sequencing typically captures a large part of the extended splice site at sufficient coverage for variant identification. However, mutations in the extended splice site are typically excluded during the prioritization step because variation within these regions is more prevalent but also more difficult to interpret. Existing algorithms for splice sites such as MaxEntScan (Eng et al. 2004) and NNSplice (Reese et al. 1997) were not designed to offer predictions for the large numbers of variants from exome sequencing (Jian et al. 2014). Like for coding variants recent developments in algorithms have improved the ability to interpret this type of variants.

The SPANR (splicing-based analysis for variants) tool is another example of a “deep learning” computational model scoring the effect of variants on the mRNA-splicing. The SPANR model is trained on 1393 sequence features extracted from 10,689 alternatively spliced exons and their corresponding mRNA expression levels in 16 human tissues and offers predictions up to 300 bp within the intron (Xiong et al. 2015). The authors found that SPANR correctly predicted the direction of change in expression of the exon for 73 out of 99 (74 %) splice site mutations. Another novel splice site prediction tool called hexamer additive linear (HAL) is a model, trained on nearly two million synthetic alternatively spliced mini genes, to predict the effect of 5′ and 3′ mutations on exon skipping (Rosenberg et al. 2015). In a set of 286 variants within three genes (*CTFR*, *BRCA2* and *SMN2*) the prediction accuracy ranged from 86 to 90 %. These improvements in splice site prediction programs may open up new avenues for the interpretation of variants in exomes.

### Gene prioritization

For the interpretation of exome data it is not sufficient to only determine whether a variant is likely to impair normal gene function, but also whether the function of a mutated gene is actually relevant for the disease (MacArthur et al. 2012). Two novel approaches for the interpretation of gene function have gained a lot of traction.

Phenotypic interpretation of variants in exomes (PHIVE) is an algorithm that computes phenotype similarity between human disease phenotypes and phenotype information from knockout experiments in model organisms. This gene-level information is then combined with variant pathogenicity predictions and thereby achieves better rankings of pathogenic variants in exome data (Robinson et al. 2014). A totally different approach to predict deleteriousness for genes is based on the use of population variation to determine how intolerant genes are to normal variation. Two studies independently showed that human disease genes are much more intolerant to genetic variation than other genes (Khurana et al. 2013; Petrovski et al. 2013). Several studies have already successfully used this approach to prioritize genes with likely pathogenic mutations (Allen et al. 2013; Gilissen et al. 2014).

Overall, algorithm development has leveraged the availability of genome-wide datasets such as exome sequencing project (ESP) (Fu et al. 2013), encyclopedia of DNA elements (ENCODE) (The ENCODE Project Consortium 2012) and the International Mouse Phenotype Consortium (IMPC) (Brown and Moore 2012) to provide improved pathogenicity predictions for variants and to cope with exome-sized variant datasets. These novel algorithms represent our first steps to the next big challenge, the interpretation of non-coding variation from whole genome sequencing experiments. In the meanwhile, technologies for high-throughput functional assays are under development that may produce the high-throughput functional validations needed to improve in silico variant predictions (Findlay et al. 2014).

## Finding recurrent mutations

Besides the interpretation of variants and genes, progress has also been made in the approaches to provide proof of pathogenicity for novel candidate genes. While functional proof of pathogenicity remains crucial, it is time-consuming and expensive to obtain, and requires specific expertise. An additional layer of evidence for pathogenicity of a mutation in a candidate disease gene can be obtained by identifying multiple patients with mutations in the same gene and a similar phenotype. Two different approaches for finding recurrently mutated candidate genes have emerged, depending on whether the disorder is either rare and monogenic or more common and genetically heterogeneous.

### Genotype-centric approach for common genetically heterogeneous disorders

For genetically heterogeneous disorders, it is not possible to select specific subsets of patients based on their phenotype to perform a targeted analysis of the candidate gene. Therefore, screening of a large cohort of patients for additional mutations within the same candidate gene is typically performed (de Ligt et al. 2012; O’Roak et al. 2012). When costs allow, it is even more efficient to immediately screen the entire cohort by exome sequencing, rather than start with a small number of selected samples (Neale et al. 2012; O’Roak et al. 2011; The Deciphering Developmental Disorders Study 2015). In such a set-up, however, the probability of random findings becomes very large and rigorous statistics are required. Statistical methods do not only protect against potential false positive findings but are also able to take into account factors like reduced penetrance, modifiers, and multigenic effects (MacArthur et al. 2014). The first large-scale exome sequencing studies already relied on different statistical approaches based on estimates of genome-wide mutation rates to identify genes enriched for de novo mutations (Neale et al. 2012; O’Roak et al. 2011). An improved statistical framework was proposed by Samocha et al. (2014) which was first applied by the DDD project which performed large-scale trio sequencing of 1133 trios with developmental disorders (The Deciphering Developmental Disorders Study 2015). After identifying de novo coding mutations in this cohort, a statistical approach was used based on estimates of the gene specific mutation rate to identify 12 novel genes that were enriched for de novo mutations. The same group also used a novel statistical framework for the identification of recessive genes in a cohort of 4125 families with developmental disorders (Akawi et al. 2015). In this case a model was constructed to estimate the probability of drawing *n* unrelated families with similar biallelic genotypes by chance from the general population. Estimates of population allele frequencies for rare loss-of-function and missense variants were obtained from the Exome Aggregation Consortium data set (Lek et al. 2015). Although in both studies the cohorts are considered to be very large, statistical power was still limited and the authors emphasize that this should motivate data-sharing through international databases.

### Phenotype-centric approach for rare monogenic disorders

For many Mendelian diseases the phenotype is very rare, and individual groups do not have more than a few cases making it impossible to perform large-scale screening. The alternative is then to take a phenotype-centric approach where one finds additional patients with the same, or similar, distinct phenotype. Once more patients have been identified with overlapping phenotypes specific testing of candidate genes can be performed. Alternatively, there is the opposite approach in which first patients with matching genotypes are identified and final evidence of pathogenicity comes from the matching of patient phenotypes. In both cases additional evidence is obtained not just by the common genotype, but also by the shared specific phenotype of patients with mutations. This approach is now facilitated by various data-sharing initiatives for rare diseases such as DECIPHER (Bragin et al. 2014), Café Variome (Lancaster et al. 2015), GeneMatcher (Sobreira et al. 2015), RD-connect (Thompson et al. 2014), and PhenomeCentral (Buske et al. 2015). See Brookes and Robinson for an overview of data-sharing initiatives and databases (Brookes and Robinson 2015). The matchmaker exchange is a recent initiative to integrate the information from all of these databases by providing a single interface for queries together with match-making algorithms (Philippakis et al. 2015). A nice example of a phenotype-centric approach is a recent paper on the identification *RSPRY1* by which the authors identified an additional case with the same phenotype using the Care4Rare Canada matchmaker (Faden et al. 2015). This should hopefully inspire more researchers to contribute to these databases and facilitate the identification of the genetic cause for their patients. By contributing these data to public databases they do not only become available to researchers and physicians but also to the patients themselves (Chong et al. 2015; Kirkpatrick et al. 2015). A nice illustration of this is the case of Massimo Damiani who suffered from an unclassified form of leukoencephalopathy and whose parent’s efforts resulted in the genetic diagnosis (Lambertson et al. 2015). The authors argue that these patient-led efforts have the potential to increase the value of matchmaking networks.

### Structured phenotypes

The probability of success for matchmaking increases with the availability of good phenotype information. A long-standing challenge with phenotype descriptions is the lack of standardization. This presents several problems such as the use of different clinical nomenclature for similar phenotypes, the uncertainty whether phenotypes are absent or not assessed, and the fact that it is unclear how phenotypes are related to each other, which makes it difficult to perform computational analyses (Kohler et al. 2014). For some years these issues have been tackled by the introduction of standardized phenotype vocabularies and ontologies. Several ontologies have been developed but one of the most used is the human phenotype ontology (HPO) (Kohler et al. 2014). HPO currently consists of more than 250,000 phenotypic annotations (Groza et al. 2015). The practical benefits of using HPO have been demonstrated by the development of novel tools that facilitate the prioritization of exome variants (as discussed in the previous section), but also by a recent study of the DDD project. Akawi et al. used structured phenotype information to statistically quantify the phenotypic similarity of patients with developmental delay for which rare mutations were identified in the same gene (Akawi et al. 2015). Although the added value of the integrated phenotypes in the statistical assessment was limited, this will likely improve when phenotype information becomes more comprehensive. Obtaining comprehensive structured phenotypes, however, is difficult and time-consuming. The DDD project mandated the availability of phenotype information in HPO format for all of their samples (Firth and Wright 2011). Such criteria are not easily imposed for most other projects and several tools have been developed to encourage and facilitate the use of phenotype information. PhenoDB (Hamosh et al. 2013) and PhenoTips (Girdea et al. 2013) are platforms that allow clinicians to enter, store and analyze structured phenotypic data. Phenominer is a tool able to extract phenotype contexts from simple text to identify relationships between human diseases described in OMIM and literature (Collier et al. 2015). In the future even the actual measuring of phenotypes may be automated leading to more robust and objective phenotypes that will also take less time of physicians to administrate (Oellrich et al. 2015), and allowing bioinformaticians to use these data for interpretation of exome variants.

## Conclusions

Here we have discussed some of ongoing bioinformatic developments that have the potential to impact the way we currently analyze and interpret exome data. It is clear that many developments in bioinformatics are still needed with respect to exome sequencing and that this is still a very active field of development. This requires a high degree of flexibility and adaptiveness from those working in this field. Especially since new challenges are already on the horizon with the anticipated large-scale application of whole genome sequencing.
